# Supplementary material for: Alpha power increases spontaneously during a neurofeedback session
Source: Commun Psychol. 2026 Mar 12;4:75. doi: 10.1038/s44271-026-00431-w (PMC13125214; doi:10.1038/s44271-026-00431-w)
Supplement: Supplementary file 2 — Supplemental Material [file 44271_2026_431_MOESM2_ESM.pdf]

Supplementary Material for:

Alpha power increases spontaneously during a neurofeedback session

Jacob Maaz,*[1,2,3] Laurent Waroquier,[4] Alexandra Dia,[1,2] Véronique Paban,[1,2] and Arnaud Rey[3]

Author affiliations:

1 Aix Marseille Univ, CNRS, CRPN, 13331 Marseille, France

2 Institute Neuro-Marseille, NeuroSchool, Aix Marseille Univ, France

3 Institute of Language, Communication and the Brain, Aix Marseille Univ, France

4 Aix Marseille Univ, PSYCLE, 13621 Aix-en-Provence, France

Correspondence to: Jacob Maaz

Centre for Research in Psychology and Neuroscience (CRPN) – UMR 7077

CNRS – Aix-Marseille Université

3, place Victor Hugo – Case D

13331 Marseille Cedex 3 – France

jacob.maaz@univ-amu.fr

Supplementary Table 1 Completed CRED-nf checklist from Ros et al.[14]

1. Pre-experiment

a. This study was preregistered via the Open Science Framework (OSF).
b. The sample size of the present study was determined using a Bayesian a priori power analysis. Results suggested that a sample size of 24 per group was sufficient to obtain enough statistical power given the present design and analyses.

2. Control groups

a. We also evaluate the effects and corresponding interactions of trial repetition with both: (i) the veracity of feedback (i.e., comparing the Genuine and Sham groups of the current study), and (ii) the engagement in self-regulation (i.e., comparing the Sham group to the independent, Passive feedback visualisation group).
b. Before data collection, random allocation to genuine EEG-NF or sham groups was performed using an in-house Matlab script. Both participants and experimenters remained blind to group assignments. This double-blinding was supported by an authorised deception protocol to minimise negative psychosocial influences (e.g., motivation, expectations).
c. Blinding of those who rate the outcome and those who analyse the data:
   - Both participants and experimenters remained blind to group assignments.
   - Group allocation remained hidden during statistical analyses.
d. To evaluate the success of the double-blind sham-controlled procedure (i.e., participants should perceive similarly the genuine and sham feedback), participants were asked at the end of the session their feeling of control over the feedback variations and how strongly they believe that these variations were actually random.
e. NA: This is not a clinical efficacy study

3. Control measures

a. Psychosocial factors were not measured
b. The following verbal instructions (in French) were given before the beginning of the session: “You will complete three blocks of neurofeedback training. Each block is composed of eight one-minute trials. During the eight trials of a block, a circle will be presented at the centre of the screen while continuously growing and decreasing in size. Depending on the block, its size will grow and decrease at different rates. At any given moment, the size of the circle reflects the state of your brainwaves in real-time, which are influenced by your thoughts. By keeping your eyes on the circle, your task is thus to try your best to increase as much as possible the size of the circle thanks to your thoughts. The goal is to find the best mental strategy that effectively renders the circle as big as possible. Over the course of the trials, you will progressively become able to self-modulate your brainwaves by intentionally modifying your thoughts and adopting the best mental strategy.”
c. The strategies participants used were not recorded or not reported in the manuscript
d. The manuscript does not report the methods used for online-data processing and artifact correction
e. Condition and group effects for artifacts were not measured, or not reported in the manuscript

4. Feedback specifications

a. For the genuine EEG-NF group, the circle size was updated as a real-time feedback of the participant’s alpha band (8-12 Hz) spectral power at Pz.
b. See Material and neurofeedback implementation, as well as Procedure sections.
c. All participants underwent an EEG-NF session comprising four blocks of eight 60-second trials. During each trial, a grey circle was presented at the centre of a screen. Within three “training” blocks, the circle size was continuously updated. Participants had to maximise the circle size as much as possible. For the genuine EEG-NF group, the circle size was updated as a real-time feedback of the participant’s alpha band (8-12 Hz) spectral power at Pz. By contrast, for the sham group, the circle size was

determined before data collection using alpha power fluctuations of participants from an independent study

d. For the genuine EEG-NF group, the circle size was updated as a real-time feedback of the participant's alpha band (8-12 Hz) spectral power at Pz[8].

e. The implementation of the EEG-NF session, as well as EEG data acquisition, online and offline processing were conducted in Matlab Release 2023a (Mathworks, Inc.). Specifically, EEG data was acquired with Brainflow library version 5-8-1, using an OpenBCI Cyton 8-channels board with OpenBCI Gold cup and Earclip electrodes. The genuine and sham feedbacks were implemented using Psychtoolbox-3.

5. Outcome measures – Brain

a. Concerning the effect of trial repetition, extreme evidence was found in favour of a positive effect on alpha power (Fz: $\beta = 0.019$, 95% CrI [0.014, 0.025], BF_{10} = 769175401465056, BF_{10+} = *Inf.*; Cz: $\beta = 0.02$, 95% CrI [0.014, 0.026], BF_{10} = 417951444524249, BF_{10+} = *Inf.*; Pz: $\beta = 0.02$, 95% CrI [0.013, 0.026], BF_{10} = 99443180922583, BF_{10+} = *Inf.*).

b. Fig. 2 presents the alpha power evolution within the training block with a 1 Hz feedback update frequency, depending on the Task to which participants were submitted (i.e., Genuine EEG-NF, Sham EEG-NF, or Passive feedback visualisation).
AND:
Fig. 5 displayed the evolution of each features considered across the whole session.

c. Importantly, extreme evidence supported the absence of interaction of trial repetition with both the veracity of feedback (Fz: $\beta = -0.001$, 95% CrI [-0.016, 0.013], BF_{10} = 0.007; Cz: $\beta = 0.004$, 95% CrI [-0.011, 0.019], BF_{10} = 0.009; Pz: $\beta = 0.002$, 95% CrI [-0.014, 0.017], BF_{10} = 0.008) and the engagement in self-regulation (Fz: $\beta = 0.003$, 95% CrI [-0.011, 0.017], BF_{10} = 0.008; Cz: $\beta = 0.003$, 95% CrI [-0.012, 0.017], BF_{10} = 0.008; Pz: $\beta = 0.005$, 95% CrI [-0.01, 0.02], BF_{10} = 0.009).

6. Outcome measures – Behaviour

a. The manuscript does not include measures of clinical or behavioural significance

b. This manuscript does not compare regulation success and behavioural outcomes

7. Data storage

a. All materials, data, analysis codes, and preregistration are available via OSF.

Supplementary Table 2 Checklist guidelines for time-frequency analyses (adapted from Keil *et al.*[69]).

#	Information to be included in the manuscript	Completed?
1	The specific stage of processing in which time-frequency analysis was applied (e.g., single-trials, after trial averaging, etc.). This clarifies which aspect(s) of oscillatory activity (e.g., spontaneous and/or induced, evoked, etc.) are being observed. If averaged potentials of each trial were subtracted prior to conducting time-frequency analyses on single trials, this step should be stated along with figures depicting the averaged potential in both time and frequency domains.	YES
2	For authors using Fourier-based time-frequency analyses (spectrograms), the following recommendations are provided for each specific approach: (1) If using spectrograms, or moving-window DFT/FFT analyses, report the specific window size and step size. Additional within-window averaging averaging achieved via algorithms (e.g., Welch periodogram method) should also be reported. (2) If using multitaper analyses, the type of tapering windows used, total number used, their center frequencies, whether any smoothing factors are applied, and the specific algorithms used to form their shapes should be reported. (3) If using complex demodulation, the frequencies examined, and the specific properties of the low-pass filter used (i.e., filter type, order, and cutoff frequency) should be reported.	YES
3	For authors conducting time-frequency analyses based on time domain filtering methods (i.e., Filter-Hilbert or similar approaches), the software and version number of the Hilbert transform used to identify the phase-shifted version of the empirical signal. In addition, authors should state the specific properties of band-pass filter (i.e., filter types, order, and cutoff frequencies).	Not applicable
4	If using wavelet-based methods for time-frequency analyses, include the smoothing/ smearing for the minimum and maximum frequency of interest and indicate the maximal temporal and frequency smoothing for a specific wavelet family. In addition, using Morlet wavelets, include the Morlet parameter (m) indicating the trade-off between time and frequency smoothing and smoothing values in the time (σt) and frequency (σf) domains.	Not applicable
5	As for frequency domain analyses, specify the duration of analytical time segments used, with pre- and post-event onset duration. In addition, include the number of time segments for each condition/group.	YES
6	Descriptions of any nonlinear transformations and/or baseline adjustment that were used prior to statistical analyses, accompanied by a rationale for these decisions. Specifically, include the duration used as a baseline and the type of algorithm (e.g., division, subtraction, etc.) used for this adjustment.	Not applicable

Supplementary Table 3 Checklist guidelines for spectral analyses (adapted from Keil *et al.*[69]).

#	Information to be included in the manuscript	Completed?
1	Specifying the inputs and outputs of all algorithms used in the processing pipeline	YES
2	A discussion of how oscillatory activity was conceptualised relative to 1/*f* noise and/or other broadband phenomena (underlying model)	YES
3	A rationale for the choice of measurement of power in a specific frequency band, including how nonperiodic (1/*f*) contributions to the spectrum were addressed	YES
4	A statement describing the specific type of Fourier- or non-Fourier-based algorithm used for transformation from the time domain to the frequency domain	YES
5	The exact duration of time segment used for transformation into the frequency domain for each condition of interest. In addition, the total number of segments (e.g., trials per condition) entering an averaged spectrum, along with how data epochs were combined within and across recordings (e.g., overlapping windows)	YES
6	The type, total number of, overlap between, and duration of any taper window functions, along with their ramp-on and ramp-off duration. If alternative and/or additional steps were taken to address edge artifacts, these should be stated. If applicable, the choice of taper window function should be specified as being guided by computational principles and/or by aiming to replicate current methods (e.g., Hann or Hamming window)	YES
7	If zero-padding is applied, the number and location of added zeros (e.g., before the time series, after the time series, or both before and after the time series)	Not applicable
8	All normalisation steps (e.g., by length of time, multiplication of the lower half of the spectrum, or by complex conjugate, etc.) applied to the spectral power or power density calculation	YES
9	The native frequency resolution of the spectrum (e.g., 1/(epoch duration in seconds)). In addition, the number of frequency bins extracted for a specific band of interest, and the range of these binds (e.g., 7.98 Hz to 11.97 Hz)	YES
10	Whether analyses were conducted using single trials or the average across trials	YES
11	How band power was measured from a spectrum	YES

Supplementary Table 4 Number of Independent Components removed from the data of each participant.

Participant's number	Number of Independent Components removed
1	2
2	3
3	2
4	2
5	2
6	2
7	3
8	2
9	2
10	2
11	2
12	2
13	2
14	3
15	2
16	2
17	2
18	2
19	2
20	2
21	2
22	2
23	2
24	2
25	3
26	2
27	1
28	2
29	2
30	3
31	2
32	2
33	2
34	2
35	3
36	3
37	2
38	2
39	2
40	3
41	2
42	2
43	1

44	3
45	1
46	2
47	2
48	2
49	1
50	2
51	3
52	2
53	2
54	3
55	3
56	3
57	2
58	3
59	3
60	1

All components were identified using the EEGLAB extended Infomax Independent Component Analysis (ICA) algorithm[71]. Independent Components for eye blinks and lateral eye movements were identified for rejection and subtracted from the data by visual inspection of the component scalp topography, time series, and power spectrum distributions. Note that, unusually, three components were removed from the data of 14 participants. This was done because the ICA algorithm split one of the two typical eye artifact components (one for eye blink and one for lateral eye movements) into two different components. For example, ICA on participant 7's data resulted in a duplication of the typical eye blink component.

Supplementary Table 5 Custom-coded repeated-contrast matrix assigned to the Frequency predictor of each model computed on training blocks.

Level labels	Intercept	5 *Hz vs.* 1 *Hz* (1st contrast)	10 *Hz vs.* 5 *Hz* (2nd contrast)
1 *Hz*	1	-2/3	-1/3
5 *Hz*	1	1/3	-1/3
10 *Hz*	1	1/3	2/3

Between the three training blocks of the present EEG-NF session, we manipulated in all groups the Frequency of feedback update: 1 *Hz*, 5 *Hz*, or 10 *Hz*. To relate to our hypotheses testing, we applied the present repeated-contrast matrix to the categorical predictor 'Frequency'. This matrix was obtained by applying the generalised inverse to a *Hypothesis* matrix referring to our hypotheses. The '5 *Hz vs.* 1 *Hz*' (1st contrast) column relates to the hypothesis that there is a difference in spectral power when participants are presented a feedback updated at 5 *Hz* relative to 1 *Hz*. The '10 *Hz vs.* 5 *Hz*' (2nd contrast) column relates to the hypothesis that there is a difference in spectral power when participants are presented a feedback updated at 10 *Hz* relative to 5 *Hz*.

Supplementary Table 6 Custom-coded contrast matrix assigned to the Task predictor of each model.

Level labels	Intercept	Genuine *vs.* Sham (1st contrast)	Sham *vs.* Passive (2nd contrast)
Genuine	1	-2/3	-1/3
Sham	1	1/3	-1/3
Passive	1	1/3	2/3

In the present study, we manipulated the veracity of the feedback presented to participants using a double-blind sham-controlled design. Participants were divided in two groups: one did a 'Genuine' and the other a 'Sham' EEG-NF session. In addition, to evaluate whether the engagement in self-regulation influences our results, we compared the present 'Sham' group to the group from previous work[31] who performs a 'Passive' visualisation task. This task was similar to the present 'Sham' session, but without explicitly engaging participants in self-regulation. To relate to our hypotheses testing of both effects, we applied the present contrast matrix to the 'Task' predictor of our models. This matrix was obtained by applying the generalised inverse to a *Hypothesis* matrix referring to ours hypotheses. The 'Genuine *vs.* Sham' (1st contrast) column relates to the hypothesis that there is a difference in spectral power when participants are presented a genuine compared to a sham feedback. The 'Sham *vs.* Passive' (2nd contrast) column relates to the hypothesis that there is a difference in spectral power when participants are engaged in self-regulating the feedback relative to when they are solely instructed to visualise it.

Supplementary Table 7 Estimates from models computed on alpha spectral power during training blocks.

Electrode	Parameter	Estimate	Lower	Upper	BF_{10}	BF_{10+}
**Fz**	**Trial**	**0,019**	**0,014**	**0,025**	**769175401465056**	***Inf.***
Fz	Frequency - 5 Hz *vs.* 1 Hz	0,029	-0,035	0,093	0.048	4.294
Fz	Frequency - 10 Hz *vs.* 5 Hz	0,018	-0,056	0,091	0.041	2.144
Fz	Task - Genuine *vs.* Sham	0,331	-0,148	0,809	0.621	10.868
Fz	Task - Sham *vs.* Passive	-0,122	-0,593	0,347	0.272	0.433
Fz	Trial:Frequency - 5 Hz *vs.* 1 Hz	-0,006	-0,018	0,005	0.011	0.145
Fz	Trial:Frequency - 10 Hz *vs.* 5 Hz	0,002	-0,011	0,015	0.007	1.707
Fz	Trial:Task - Genuine *vs.* Sham	-0,001	-0,016	0,013	0.007	0.78
Fz	Trial:Task - Sham *vs.* Passive	0,003	-0,011	0,017	0.008	1.869
Fz	Frequency - 5 Hz *vs.* 1 Hz:Task - Genuine *vs.* Sham	0,001	-0,156	0,158	0.08	1.02
Fz	Frequency - 10 Hz *vs.* 5 Hz:Task - Genuine *vs.* Sham	-0,065	-0,246	0,117	0.117	0.314
Fz	Frequency - 5 Hz *vs.* 1 Hz:Task - Sham *vs.* Passive	-0,052	-0,207	0,103	0.098	0.34
Fz	Frequency - 10 Hz *vs.* 5 Hz:Task - Sham *vs.* Passive	0,026	-0,153	0,204	0.094	1.597
Fz	Trial:Frequency - 5 Hz *vs.* 1 Hz:Task - Genuine *vs.* Sham	0,004	-0,024	0,031	0.014	1.527
Fz	Trial:Frequency - 10 Hz *vs.* 5 Hz:Task - Genuine *vs.* Sham	-0,002	-0,035	0,03	0.017	0.813
Fz	Trial:Frequency - 5 Hz *vs.* 1 Hz:Task - Sham *vs.* Passive	-0,006	-0,033	0,021	0.015	0.486
Fz	Trial:Frequency - 10 Hz *vs.* 5 Hz:Task - Sham *vs.* Passive	0,005	-0,027	0,038	0.017	1.691
**Cz**	**Trial**	**0,02**	**0,014**	**0,026**	**417951444524249**	***Inf.***
Cz	Frequency - 5 Hz *vs.* 1 Hz	0,032	-0,037	0,1	0.054	4.659
Cz	Frequency - 10 Hz *vs.* 5 Hz	-0,002	-0,083	0,079	0.041	0.935
Cz	Task - Genuine *vs.* Sham	0,441	-0,029	0,909	1.36	32.18
Cz	Task - Sham *vs.* Passive	-0,182	-0,642	0,274	0.314	0.271
Cz	Trial:Frequency - 5 Hz *vs.* 1 Hz	-0,007	-0,019	0,005	0.012	0.136
Cz	Trial:Frequency - 10 Hz *vs.* 5 Hz	0,002	-0,012	0,016	0.007	1.61
Cz	Trial:Task - Genuine *vs.* Sham	0,004	-0,011	0,019	0.009	2.23
Cz	Trial:Task - Sham *vs.* Passive	0,003	-0,012	0,017	0.008	1.719
Cz	Frequency - 5 Hz *vs.* 1 Hz:Task - Genuine *vs.* Sham	-0,054	-0,222	0,113	0.106	0.354
Cz	Frequency - 10 Hz *vs.* 5 Hz:Task - Genuine *vs.* Sham	-0,083	-0,283	0,115	0.142	0.254
Cz	Frequency - 5 Hz *vs.* 1 Hz:Task - Sham *vs.* Passive	-0,036	-0,201	0,129	0.091	0.495
Cz	Frequency - 10 Hz *vs.* 5 Hz:Task - Sham *vs.* Passive	0,04	-0,156	0,236	0.108	1.926
Cz	Trial:Frequency - 5 Hz *vs.* 1 Hz:Task - Genuine *vs.* Sham	0,005	-0,024	0,035	0.016	1.747
Cz	Trial:Frequency - 10 Hz *vs.* 5 Hz:Task - Genuine *vs.* Sham	-0,006	-0,041	0,029	0.019	0.568
Cz	Trial:Frequency - 5 Hz *vs.* 1 Hz:Task - Sham *vs.* Passive	-0,007	-0,036	0,023	0.016	0.482
Cz	Trial:Frequency - 10 Hz *vs.* 5 Hz:Task - Sham *vs.* Passive	0,005	-0,029	0,04	0.018	1.651

**Pz**	**Trial**	**0,02**	**0,013**	**0,026**	**99443180922582.8**	***Inf.***
Pz	Frequency - 5 Hz *vs.* 1 Hz	0,011	-0,059	0,082	0.037	1.669
Pz	Frequency - 10 Hz *vs.* 5 Hz	-0,036	-0,115	0,044	0.06	0.23
Pz	Task - Genuine *vs.* Sham	0,512	0,056	0,968	2.662	73.391
Pz	Task - Sham *vs.* Passive	-0,211	-0,655	0,236	0.354	0.212
Pz	Trial:Frequency - 5 Hz *vs.* 1 Hz	-0,009	-0,021	0,003	0.02	0.065
Pz	Trial:Frequency - 10 Hz *vs.* 5 Hz	0,004	-0,008	0,017	0.008	3.157
Pz	Trial:Task - Genuine *vs.* Sham	0,002	-0,014	0,017	0.008	1.371
Pz	Trial:Task - Sham *vs.* Passive	0,005	-0,01	0,02	0.009	2.787
Pz	Frequency - 5 Hz *vs.* 1 Hz:Task - Genuine *vs.* Sham	-0,123	-0,297	0,049	0.24	0.087
Pz	Frequency - 10 Hz *vs.* 5 Hz:Task - Genuine *vs.* Sham	-0,009	-0,205	0,187	0.1	0.865
Pz	Frequency - 5 Hz *vs.* 1 Hz:Task - Sham *vs.* Passive	0,039	-0,131	0,209	0.096	2.089
Pz	Frequency - 10 Hz *vs.* 5 Hz:Task - Sham *vs.* Passive	0,089	-0,106	0,282	0.149	4.499
Pz	Trial:Frequency - 5 Hz *vs.* 1 Hz:Task - Genuine *vs.* Sham	0,013	-0,016	0,042	0.022	4.439
Pz	Trial:Frequency - 10 Hz *vs.* 5 Hz:Task - Genuine *vs.* Sham	-0,015	-0,046	0,015	0.025	0.196
Pz	Trial:Frequency - 5 Hz *vs.* 1 Hz:Task - Sham *vs.* Passive	-0,014	-0,043	0,014	0.023	0.197
Pz	Trial:Frequency - 10 Hz *vs.* 5 Hz:Task - Sham *vs.* Passive	0,001	-0,029	0,031	0.015	1.157

Each model reported has been computed twice in order to ensure the stability of the BFs. If not specified, each numerical value corresponds to the average of the values obtained across these two model computations. The 'Estimate' column stands for the averaged posterior group-level effects (slopes) of each model 'Parameter' (in z-score standardised units). For the 'Trial' predictor, the estimate corresponds to the group-level effect of one trial of the 1 *Hz* training block (defined as reference for subsequent comparisons for the Frequency predictor) of the Genuine group (defined as reference for subsequent comparisons for the Task predictor). For the 'Frequency' predictor, each comparison (i.e., '5 *Hz vs.* 1 *Hz*' and '10 *Hz vs.* 5 *Hz*') estimate refers to the group-level effect during each training block first trial (modality of Trial predictor defined as reference for subsequent comparisons) of the Genuine group. For the 'Task' predictor, the estimate of both comparisons ('Genuine *vs.* Sham' and 'Sham *vs.* Passive') refers to the between-group effect within the first trial of the 1 *Hz* training block. The 'Lower' and 'Upper' columns correspond to the minimal lower and maximal upper bounds of the two 95% CrI computed. The 'BF_{10}' and 'BF_{10+}' columns correspond to the BF in favour of the alternative hypothesis (relative to the null) and the positive directional (i.e., one-sided) BF, respectively.
Lines in gold highlight corresponding electrode and parameter for which BFs quantify sufficient evidence in favour of the alternative hypothesis over the null (i.e., presence of an effect) on alpha power.

Supplementary Table 8 Estimates from models computed on alpha spectral power during the transfer block.

Electrode	Parameter	Estimate	Lower	Upper	BF_{10}	BF_{10+}
**Fz**	**Trial**	**0,033**	**0,022**	**0,043**	**1322458781605221**	***Inf.***
Fz	Task - Genuine *vs.* Sham	0,338	-0,155	0,832	0.635	10.574
Fz	Task - Sham *vs.* Passive	-0,219	-0,697	0,261	0.365	0.223
Fz	Trial:Task - Genuine *vs.* Sham	0,012	-0,014	0,038	0.02	4.71
Fz	Trial:Task - Sham *vs.* Passive	-0,031	-0,056	-0,006	0.248	0.008
**Cz**	**Trial**	**0,034**	**0,024**	**0,045**	**645504366446923**	***Inf.***

Cz	Task - Genuine *vs.* Sham	0,468	-0,019	0,95	1.516	34.547
Cz	Task - Sham *vs.* Passive	-0,284	-0,76	0,192	0.482	0.134
Cz	Trial:Task - Genuine *vs.* Sham	0,013	-0,014	0,04	0.022	5.164
Cz	Trial:Task - Sham *vs.* Passive	-0,027	-0,054	-0,001	0.107	0.022
**Pz**	**Trial**	**0,03**	**0,02**	**0,041**	**61337222253310**	***Inf.***
Pz	Task - Genuine *vs.* Sham	0,549	0,062	1,033	2.835	72.043
Pz	Task - Sham *vs.* Passive	-0,402	-0,891	0,084	0.964	0.051
Pz	Trial:Task - Genuine *vs.* Sham	0,014	-0,013	0,041	0.023	5.463
Pz	Trial:Task - Sham *vs.* Passive	-0,013	-0,04	0,013	0.022	0.189

Each model reported has been computed twice in order to ensure the stability of the BFs. If not specified, each numerical value corresponds to the average of the values obtained across these two model computations. The 'Estimate' column stands for the averaged posterior group-level effects (slopes) of each model 'Parameter' (in z-score standardised units). For the 'Trial' predictor, the estimate corresponds to the group-level effect of one trial within the Genuine group (defined as reference for subsequent comparisons for the Task predictor). For the 'Task' predictor, the estimate of both comparisons ('Genuine *vs.* Sham' and 'Sham *vs.* Passive') refers to the between-group effect within the first trial of the transfer block (defined as reference for subsequent comparisons for the Trial predictor). The 'Lower' and 'Upper' columns correspond to the minimal lower and maximal upper bounds of the two 95% CrI computed. The 'BF_{10}' and 'BF_{10+}' columns correspond to the BF in favour of the alternative hypothesis (relative to the null) and the positive directional (i.e., one-sided) BF, respectively.
Lines in gold highlight corresponding electrode and parameter for which BFs quantify sufficient evidence in favour of the alternative hypothesis over the null (i.e., presence of an effect) on alpha power.

Supplementary Table 9 Estimates from models computed on theta, SMR and beta spectral power during training blocks.

Frequency Band	Electrode	Predictor	Estimate	Lower	Upper	BF_{10}	BF_{10+}
**Theta**	**Fz**	**Trial**	**0,014**	**0,007**	**0,02**	**9.146**	**16968,697**
Theta	Fz	Frequency - 5 Hz *vs.* 1 Hz	0,053	-0,021	0,126	0.104	12.19
Theta	Fz	Frequency - 10 Hz *vs.* 5 Hz	-0,049	-0,119	0,021	0.093	0.09
Theta	Fz	Task - Genuine *vs.* Sham	0,187	-0,284	0,656	0.323	3.667
Theta	Fz	Task - Sham *vs.* Passive	-0,031	-0,49	0,435	0.235	0.806
Theta	Fz	Trial:Frequency - 5 Hz *vs.* 1 Hz	-0,007	-0,02	0,006	0.011	0.183
Theta	Fz	Trial:Frequency - 10 Hz *vs.* 5 Hz	0,002	-0,011	0,015	0.007	1.527
Theta	Fz	Trial:Task - Genuine *vs.* Sham	0,015	-0,002	0,031	0.039	23.527
Theta	Fz	Trial:Task - Sham *vs.* Passive	-0,016	-0,032	0	0.055	0.026
Theta	Fz	Frequency - 5 Hz *vs.* 1 Hz:Task - Genuine *vs.* Sham	0,096	-0,083	0,275	0.159	5.955
Theta	Fz	Frequency - 10 Hz *vs.* 5 Hz:Task - Genuine *vs.* Sham	-0,128	-0,3	0,046	0.255	0.078
Theta	Fz	Frequency - 5 Hz *vs.* 1 Hz:Task - Sham *vs.* Passive	-0,133	-0,309	0,043	0.27	0.073
Theta	Fz	Frequency - 10 Hz *vs.* 5 Hz:Task - Sham *vs.* Passive	0,197	0,027	0,367	1.159	86.006
Theta	Fz	Trial:Frequency - 5 Hz *vs.* 1 Hz:Task - Genuine *vs.* Sham	-0,017	-0,05	0,016	0.028	0.186
Theta	Fz	Trial:Frequency - 10 Hz *vs.* 5 Hz:Task - Genuine *vs.* Sham	0,02	-0,012	0,052	0.034	8.072
Theta	Fz	Trial:Frequency - 5 Hz *vs.* 1 Hz:Task - Sham *vs.* Passive	0,029	-0,003	0,061	0.076	24.019

Theta	Fz	Trial:Frequency - 10 Hz *vs.* 5 Hz:Task - Sham *vs.* Passive	-0,04	-0,071	-0,009	0.374	0.006
SMR	Fz	Trial	0,011	0,004	0,017	0.518	> 100
SMR	Fz	Frequency - 5 Hz *vs.* 1 Hz	0,005	-0,065	0,074	0.036	1.256
SMR	Fz	Frequency - 10 Hz *vs.* 5 Hz	0,067	-0,002	0,136	0.22	34.996
SMR	Fz	Task - Genuine *vs.* Sham	0,077	-0,427	0,574	0.265	1.639
SMR	Fz	Task - Sham *vs.* Passive	-0,012	-0,499	0,482	0.245	0.926
SMR	Fz	Trial:Frequency - 5 Hz *vs.* 1 Hz	0,002	-0,011	0,016	0.007	1.75
SMR	Fz	Trial:Frequency - 10 Hz *vs.* 5 Hz	-0,006	-0,019	0,007	0.01	0.206
SMR	Fz	Trial:Task - Genuine *vs.* Sham	-0,001	-0,017	0,015	0.008	0.809
SMR	Fz	Trial:Task - Sham *vs.* Passive	-0,003	-0,018	0,013	0.008	0.577
SMR	Fz	Frequency - 5 Hz *vs.* 1 Hz:Task - Genuine *vs.* Sham	0,135	-0,036	0,306	0.293	15.787
SMR	Fz	Frequency - 10 Hz *vs.* 5 Hz:Task - Genuine *vs.* Sham	-0,071	-0,24	0,098	0.119	0.256
**SMR**	**Fz**	**Frequency - 5 Hz *vs.* 1 Hz:Task - Sham *vs.* Passive**	**-0,237**	**-0,405**	**-0,068**	**3.713**	**0.003**
SMR	Fz	Frequency - 10 Hz *vs.* 5 Hz:Task - Sham *vs.* Passive	-0,006	-0,172	0,16	0.084	0.887
SMR	Fz	Trial:Frequency - 5 Hz *vs.* 1 Hz:Task - Genuine *vs.* Sham	-0,008	-0,042	0,027	0.019	0.483
SMR	Fz	Trial:Frequency - 10 Hz *vs.* 5 Hz:Task - Genuine *vs.* Sham	0,002	-0,029	0,034	0.016	1.213
SMR	Fz	Trial:Frequency - 5 Hz *vs.* 1 Hz:Task - Sham *vs.* Passive	0,026	-0,008	0,06	0.053	13.756
SMR	Fz	Trial:Frequency - 10 Hz *vs.* 5 Hz:Task - Sham *vs.* Passive	0,002	-0,029	0,033	0.016	1.234
Beta	Fz	Trial	0,011	0,002	0,02	0.104	> 100
Beta	Fz	Frequency - 5 Hz *vs.* 1 Hz	0,007	-0,093	0,106	0.05	1.236
Beta	Fz	Frequency - 10 Hz *vs.* 5 Hz	-0,025	-0,115	0,065	0.052	0.415
Beta	Fz	Task - Genuine *vs.* Sham	0,016	-0,496	0,524	0.255	1.116
Beta	Fz	Task - Sham *vs.* Passive	-0,048	-0,548	0,455	0.255	0.737
Beta	Fz	Trial:Frequency - 5 Hz *vs.* 1 Hz	-0,004	-0,027	0,019	0.012	0.584
Beta	Fz	Trial:Frequency - 10 Hz *vs.* 5 Hz	0,011	-0,006	0,028	0.02	9.514
Beta	Fz	Trial:Task - Genuine *vs.* Sham	-0,001	-0,022	0,02	0.011	0.835
Beta	Fz	Trial:Task - Sham *vs.* Passive	0,011	-0,01	0,032	0.018	5.619
Beta	Fz	Frequency - 5 Hz *vs.* 1 Hz:Task - Genuine *vs.* Sham	0,075	-0,168	0,316	0.147	2.695
Beta	Fz	Frequency - 10 Hz *vs.* 5 Hz:Task - Genuine *vs.* Sham	-0,009	-0,229	0,212	0.11	0.877
Beta	Fz	Frequency - 5 Hz *vs.* 1 Hz:Task - Sham *vs.* Passive	-0,126	-0,366	0,118	0.209	0.175
Beta	Fz	Frequency - 10 Hz *vs.* 5 Hz:Task - Sham *vs.* Passive	0,16	-0,058	0,377	0.32	12.637
Beta	Fz	Trial:Frequency - 5 Hz *vs.* 1 Hz:Task - Genuine *vs.* Sham	-0,009	-0,064	0,047	0.03	0.601
Beta	Fz	Trial:Frequency - 10 Hz *vs.* 5 Hz:Task - Genuine *vs.* Sham	0,007	-0,035	0,049	0.022	1.717
Beta	Fz	Trial:Frequency - 5 Hz *vs.* 1 Hz:Task - Sham *vs.* Passive	0,009	-0,047	0,063	0.029	1.632

Beta	Fz	Trial:Frequency - 10 Hz *vs.* 5 Hz:Task - Sham *vs.* Passive	-0,015	-0,056	0,027	0.027	0.313
Theta	Cz	Trial	0,012	0,005	0,02	0.483	> 100
Theta	Cz	Frequency - 5 Hz *vs.* 1 Hz	0,081	0,005	0,157	0.353	55.021
Theta	Cz	Frequency - 10 Hz *vs.* 5 Hz	-0,064	-0,141	0,013	0.15	0.053
Theta	Cz	Task - Genuine *vs.* Sham	0,235	-0,218	0,691	0.383	5.485
Theta	Cz	Task - Sham *vs.* Passive	-0,053	-0,5	0,399	0.232	0.686
Theta	Cz	Trial:Frequency - 5 Hz *vs.* 1 Hz	-0,011	-0,025	0,002	0.027	0.05
Theta	Cz	Trial:Frequency - 10 Hz *vs.* 5 Hz	0,003	-0,01	0,017	0.008	2.226
Theta	Cz	Trial:Task - Genuine *vs.* Sham	0,017	-0,001	0,036	0.049	29.137
Theta	Cz	Trial:Task - Sham *vs.* Passive	-0,019	-0,037	0	0.069	0.023
Theta	Cz	Frequency - 5 Hz *vs.* 1 Hz:Task - Genuine *vs.* Sham	0,069	-0,117	0,255	0.123	3.31
Theta	Cz	Frequency - 10 Hz *vs.* 5 Hz:Task - Genuine *vs.* Sham	-0,123	-0,313	0,066	0.219	0.111
Theta	Cz	Frequency - 5 Hz *vs.* 1 Hz:Task - Sham *vs.* Passive	-0,104	-0,288	0,079	0.173	0.154
Theta	Cz	Frequency - 10 Hz *vs.* 5 Hz:Task - Sham *vs.* Passive	0,182	-0,005	0,37	0.6	35.395
Theta	Cz	Trial:Frequency - 5 Hz *vs.* 1 Hz:Task - Genuine *vs.* Sham	-0,022	-0,055	0,011	0.04	0.107
Theta	Cz	Trial:Frequency - 10 Hz *vs.* 5 Hz:Task - Genuine *vs.* Sham	0,02	-0,014	0,054	0.034	6.968
Theta	Cz	Trial:Frequency - 5 Hz *vs.* 1 Hz:Task - Sham *vs.* Passive	0,035	0,002	0,068	0.144	51.192
Theta	Cz	Trial:Frequency - 10 Hz *vs.* 5 Hz:Task - Sham *vs.* Passive	-0,044	-0,078	-0,01	0.446	0.005
SMR	Cz	Trial	0,007	0,001	0,014	0.041	83.138
SMR	Cz	Frequency - 5 Hz *vs.* 1 Hz	0,022	-0,041	0,085	0.04	3.084
SMR	Cz	Frequency - 10 Hz *vs.* 5 Hz	0,084	0,018	0,149	0.773	> 100
SMR	Cz	Task - Genuine *vs.* Sham	0,132	-0,368	0,63	0.291	2.327
SMR	Cz	Task - Sham *vs.* Passive	-0,041	-0,533	0,453	0.25	0.771
SMR	Cz	Trial:Frequency - 5 Hz *vs.* 1 Hz	-0,001	-0,014	0,013	0.007	0.859
SMR	Cz	Trial:Frequency - 10 Hz *vs.* 5 Hz	-0,007	-0,02	0,006	0.012	0.156
SMR	Cz	Trial:Task - Genuine *vs.* Sham	-0,001	-0,016	0,015	0.008	0.882
SMR	Cz	Trial:Task - Sham *vs.* Passive	-0,004	-0,02	0,011	0.009	0.427
SMR	Cz	Frequency - 5 Hz *vs.* 1 Hz:Task - Genuine *vs.* Sham	0,105	-0,051	0,259	0.192	9.927
SMR	Cz	Frequency - 10 Hz *vs.* 5 Hz:Task - Genuine *vs.* Sham	-0,071	-0,233	0,09	0.12	0.235
SMR	Cz	Frequency - 5 Hz *vs.* 1 Hz:Task - Sham *vs.* Passive	-0,201	-0,354	-0,048	2.126	0.005
SMR	Cz	Frequency - 10 Hz *vs.* 5 Hz:Task - Sham *vs.* Passive	-0,008	-0,167	0,151	0.082	0.841
SMR	Cz	Trial:Frequency - 5 Hz *vs.* 1 Hz:Task - Genuine *vs.* Sham	-0,012	-0,046	0,022	0.022	0.313
SMR	Cz	Trial:Frequency - 10 Hz *vs.* 5 Hz:Task - Genuine *vs.* Sham	0,005	-0,027	0,036	0.016	1.633
SMR	Cz	Trial:Frequency - 5 Hz *vs.* 1 Hz:Task - Sham *vs.* Passive	0,025	-0,009	0,058	0.049	13.088

SMR	Cz	Trial:Frequency - 10 Hz *vs.* 5 Hz:Task - Sham *vs.* Passive	-0,009	-0,04	0,022	0.019	0.408
Beta	Cz	Trial	0,002	-0,006	0,009	0.004	1.983
Beta	Cz	Frequency - 5 Hz *vs.* 1 Hz	0,026	-0,053	0,106	0.049	2.879
Beta	Cz	Frequency - 10 Hz *vs.* 5 Hz	0,029	-0,047	0,106	0.051	3.445
Beta	Cz	Task - Genuine *vs.* Sham	0,03	-0,472	0,536	0.256	1.211
Beta	Cz	Task - Sham *vs.* Passive	-0,018	-0,512	0,477	0.252	0.886
Beta	Cz	Trial:Frequency - 5 Hz *vs.* 1 Hz	-0,008	-0,026	0,011	0.013	0.256
Beta	Cz	Trial:Frequency - 10 Hz *vs.* 5 Hz	0,004	-0,011	0,02	0.009	2.483
Beta	Cz	Trial:Task - Genuine *vs.* Sham	0,002	-0,016	0,019	0.009	1.35
Beta	Cz	Trial:Task - Sham *vs.* Passive	0,001	-0,016	0,019	0.009	1.293
Beta	Cz	Frequency - 5 Hz *vs.* 1 Hz:Task - Genuine *vs.* Sham	0,036	-0,159	0,231	0.105	1.801
Beta	Cz	Frequency - 10 Hz *vs.* 5 Hz:Task - Genuine *vs.* Sham	0,091	-0,098	0,28	0.151	4.828
Beta	Cz	Frequency - 5 Hz *vs.* 1 Hz:Task - Sham *vs.* Passive	-0,156	-0,347	0,036	0.347	0.057
Beta	Cz	Frequency - 10 Hz *vs.* 5 Hz:Task - Sham *vs.* Passive	0,082	-0,105	0,267	0.139	4.191
Beta	Cz	Trial:Frequency - 5 Hz *vs.* 1 Hz:Task - Genuine *vs.* Sham	-0,018	-0,063	0,026	0.031	0.262
Beta	Cz	Trial:Frequency - 10 Hz *vs.* 5 Hz:Task - Genuine *vs.* Sham	-0,002	-0,039	0,036	0.019	0.875
Beta	Cz	Trial:Frequency - 5 Hz *vs.* 1 Hz:Task - Sham *vs.* Passive	0,022	-0,022	0,066	0.036	5.109
Beta	Cz	Trial:Frequency - 10 Hz *vs.* 5 Hz:Task - Sham *vs.* Passive	-0,016	-0,053	0,021	0.028	0.238
Theta	Pz	Trial	0,013	0,005	0,021	0.763	1615.162
Theta	Pz	Frequency - 5 Hz *vs.* 1 Hz	0,07	-0,006	0,145	0.201	27.224
Theta	Pz	Frequency - 10 Hz *vs.* 5 Hz	-0,097	-0,181	-0,012	0.546	0.013
Theta	Pz	Task - Genuine *vs.* Sham	0,244	-0,192	0,688	0.401	6.381
Theta	Pz	Task - Sham *vs.* Passive	-0,039	-0,477	0,397	0.22	0.758
Theta	Pz	Trial:Frequency - 5 Hz *vs.* 1 Hz	-0,013	-0,028	0,001	0.04	0.034
Theta	Pz	Trial:Frequency - 10 Hz *vs.* 5 Hz	0,006	-0,01	0,021	0.01	3.223
Theta	Pz	Trial:Task - Genuine *vs.* Sham	0,011	-0,009	0,03	0.018	6.231
Theta	Pz	Trial:Task - Sham *vs.* Passive	-0,016	-0,035	0,003	0.036	0.056
Theta	Pz	Frequency - 5 Hz *vs.* 1 Hz:Task - Genuine *vs.* Sham	0,007	-0,179	0,193	0.094	1.12
Theta	Pz	Frequency - 10 Hz *vs.* 5 Hz:Task - Genuine *vs.* Sham	-0,105	-0,313	0,102	0.173	0.187
Theta	Pz	Frequency - 5 Hz *vs.* 1 Hz:Task - Sham *vs.* Passive	-0,114	-0,297	0,071	0.198	0.124
Theta	Pz	Frequency - 10 Hz *vs.* 5 Hz:Task - Sham *vs.* Passive	0,144	-0,061	0,347	0.276	11.14
Theta	Pz	Trial:Frequency - 5 Hz *vs.* 1 Hz:Task - Genuine *vs.* Sham	-0,019	-0,055	0,016	0.031	0.163
Theta	Pz	Trial:Frequency - 10 Hz *vs.* 5 Hz:Task - Genuine *vs.* Sham	0,02	-0,019	0,058	0.032	5.366
Theta	Pz	Trial:Frequency - 5 Hz *vs.* 1 Hz:Task - Sham *vs.* Passive	0,039	0,004	0,074	0.196	69.967

Theta	Pz	Trial:Frequency - 10 Hz *vs.* 5 Hz:Task - Sham *vs.* Passive	-0,043	-0,081	-0,005	0.216	0.013
SMR	Pz	Trial	0,006	0	0,011	0.017	33.094
SMR	Pz	Frequency - 5 Hz *vs.* 1 Hz	0,03	-0,026	0,085	0.05	6.099
SMR	Pz	Frequency - 10 Hz *vs.* 5 Hz	0,064	0,003	0,126	0.254	47.459
SMR	Pz	Task - Genuine *vs.* Sham	0,221	-0,278	0,716	0.374	4.323
SMR	Pz	Task - Sham *vs.* Passive	-0,088	-0,578	0,405	0.262	0.566
SMR	Pz	Trial:Frequency - 5 Hz *vs.* 1 Hz	-0,007	-0,019	0,006	0.011	0.185
SMR	Pz	Trial:Frequency - 10 Hz *vs.* 5 Hz	-0,002	-0,014	0,01	0.006	0.656
SMR	Pz	Trial:Task - Genuine *vs.* Sham	-0,007	-0,021	0,007	0.012	0.183
SMR	Pz	Trial:Task - Sham *vs.* Passive	0	-0,014	0,014	0.007	1.02
SMR	Pz	Frequency - 5 Hz *vs.* 1 Hz:Task - Genuine *vs.* Sham	0,093	-0,044	0,229	0.169	10.208
SMR	Pz	Frequency - 10 Hz *vs.* 5 Hz:Task - Genuine *vs.* Sham	-0,013	-0,167	0,139	0.078	0.766
SMR	Pz	Frequency - 5 Hz *vs.* 1 Hz:Task - Sham *vs.* Passive	-0,147	-0,281	-0,012	0.681	0.016
SMR	Pz	Frequency - 10 Hz *vs.* 5 Hz:Task - Sham *vs.* Passive	-0,07	-0,22	0,08	0.117	0.214
SMR	Pz	Trial:Frequency - 5 Hz *vs.* 1 Hz:Task - Genuine *vs.* Sham	-0,02	-0,051	0,012	0.035	0.118
SMR	Pz	Trial:Frequency - 10 Hz *vs.* 5 Hz:Task - Genuine *vs.* Sham	-0,003	-0,033	0,026	0.015	0.702
SMR	Pz	Trial:Frequency - 5 Hz *vs.* 1 Hz:Task - Sham *vs.* Passive	0,018	-0,013	0,049	0.031	7.218
SMR	Pz	Trial:Frequency - 10 Hz *vs.* 5 Hz:Task - Sham *vs.* Passive	0	-0,029	0,03	0.015	1.036
Beta	Pz	Trial	-0,007	-0,014	0,001	0.018	0.042
Beta	Pz	Frequency - 5 Hz *vs.* 1 Hz	0,049	-0,023	0,121	0.091	10.4
Beta	Pz	Frequency - 10 Hz *vs.* 5 Hz	0,023	-0,064	0,111	0.05	2.362
Beta	Pz	Task - Genuine *vs.* Sham	-0,031	-0,543	0,473	0.257	0.835
Beta	Pz	Task - Sham *vs.* Passive	0,02	-0,476	0,511	0.248	1.137
Beta	Pz	Trial:Frequency - 5 Hz *vs.* 1 Hz	-0,011	-0,028	0,006	0.02	0.107
Beta	Pz	Trial:Frequency - 10 Hz *vs.* 5 Hz	0,006	-0,011	0,023	0.011	3.064
Beta	Pz	Trial:Task - Genuine *vs.* Sham	0	-0,019	0,019	0.009	1.006
Beta	Pz	Trial:Task - Sham *vs.* Passive	0,002	-0,017	0,02	0.01	1.366
Beta	Pz	Frequency - 5 Hz *vs.* 1 Hz:Task - Genuine *vs.* Sham	-0,034	-0,211	0,143	0.097	0.544
Beta	Pz	Frequency - 10 Hz *vs.* 5 Hz:Task - Genuine *vs.* Sham	0,127	-0,089	0,342	0.216	7.247
Beta	Pz	Frequency - 5 Hz *vs.* 1 Hz:Task - Sham *vs.* Passive	-0,106	-0,282	0,069	0.182	0.128
Beta	Pz	Frequency - 10 Hz *vs.* 5 Hz:Task - Sham *vs.* Passive	0,063	-0,148	0,277	0.128	2.609
Beta	Pz	Trial:Frequency - 5 Hz *vs.* 1 Hz:Task - Genuine *vs.* Sham	-0,011	-0,053	0,03	0.024	0.419
Beta	Pz	Trial:Frequency - 10 Hz *vs.* 5 Hz:Task - Genuine *vs.* Sham	-0,01	-0,051	0,032	0.023	0.47
Beta	Pz	Trial:Frequency - 5 Hz *vs.* 1 Hz:Task - Sham *vs.* Passive	0,001	-0,04	0,042	0.021	1.085

Beta	Pz	Trial:Frequency - 10 Hz *vs.* 5 Hz:Task - Sham *vs.* Passive	-0,015	-0,056	0,025	0.027	0.297

Each model reported has been computed twice in order to ensure the stability of the BFs. If not specified, each numerical value corresponds to the average of the values obtained across these two model computations. The 'Estimate' column stands for the averaged posterior group-level effects (slopes) of each model 'Parameter' (in z-score standardised units). For the 'Trial' predictor, the estimate corresponds to the group-level effect of one trial of the 1 *Hz* training block (defined as reference for subsequent comparisons for the Frequency predictor) of the Genuine group (defined as reference for subsequent comparisons for the Task predictor). For the 'Frequency' predictor, each comparison (i.e., '5 *Hz vs.* 1 *Hz*' and '10 *Hz vs.* 5 *Hz*') estimate refers to the group-level effect during each training block first trial (modality of Trial predictor defined as reference for subsequent comparisons) of the Genuine group. For the 'Task' predictor, the estimate of both comparisons ('Genuine *vs.* Sham' and 'Sham *vs.* Passive') refers to the between-group effect within the first trial of the 1 *Hz* training block. The 'Lower' and 'Upper' columns correspond to the minimal lower and maximal upper bounds of the two 95% CrI computed. The 'BF_{10}' and 'BF_{10+}' columns correspond to the BF in favour of the alternative hypothesis (relative to the null) and the positive directional (i.e., one-sided) BF, respectively.
Lines in gold highlight the EEG features for which BFs quantify sufficient evidence in favour of the alternative hypothesis over the null (i.e., presence of an effect).

Supplementary Table 10 Estimates from models computed on theta, SMR and beta spectral power during the transfer block.

**Frequency Band**	**Electrode**	**Predictor**	**Estimate**	**Lower**	**Upper**	BF_{10}	BF_{10+}
**Theta**	**Fz**	**Trial**	**0,029**	**0,018**	**0,041**	**49519876393386**	***Inf.***
Theta	Fz	Task - Genuine *vs.* Sham	0,224	-0,279	0,723	0.381	4.334
Theta	Fz	Task - Sham *vs.* Passive	-0,126	-0,614	0,367	0.281	0.44
Theta	Fz	Trial:Task - Genuine *vs.* Sham	0,029	0,001	0,057	0.106	42.886
Theta	Fz	Trial:Task - Sham *vs.* Passive	-0,031	-0,059	-0,004	0.167	0.013
**SMR**	**Fz**	**Trial**	**0,025**	**0,014**	**0,036**	**33.727**	***Inf.***
SMR	Fz	Task - Genuine *vs.* Sham	0,031	-0,449	0,509	0.243	1.235
SMR	Fz	Task - Sham *vs.* Passive	0,006	-0,464	0,475	0.238	1.042
SMR	Fz	Trial:Task - Genuine *vs.* Sham	0,032	0,005	0,06	0.2	95
SMR	Fz	Trial:Task - Sham *vs.* Passive	-0,033	-0,06	-0,006	0.247	0.008
Beta	Fz	Trial	0,022	0,003	0,041	0.121	76.645
Beta	Fz	Task - Genuine *vs.* Sham	-0,04	-0,476	0,393	0.224	0.74
Beta	Fz	Task - Sham *vs.* Passive	0,048	-0,382	0,476	0.223	1.426
Beta	Fz	Trial:Task - Genuine *vs.* Sham	0,023	-0,024	0,07	0.038	5.182
Beta	Fz	Trial:Task - Sham *vs.* Passive	-0,016	-0,063	0,031	0.03	0.324
**Theta**	**Cz**	**Trial**	**0,028**	**0,016**	**0,041**	**60.8**	**79999**
Theta	Cz	Task - Genuine *vs.* Sham	0,278	-0,225	0,782	0.461	6.253
Theta	Cz	Task - Sham *vs.* Passive	-0,148	-0,649	0,352	0.296	0.382
Theta	Cz	Trial:Task - Genuine *vs.* Sham	0,027	-0,003	0,058	0.074	23.998
Theta	Cz	Trial:Task - Sham *vs.* Passive	-0,032	-0,062	-0,001	0.126	0.02
**SMR**	**Cz**	**Trial**	**0,018**	**0,009**	**0,027**	**7.87**	**13332.33**
SMR	Cz	Task - Genuine *vs.* Sham	0,114	-0,365	0,597	0.27	2.14
SMR	Cz	Task - Sham *vs.* Passive	-0,026	-0,493	0,443	0.241	0.84

SMR	Cz	Trial:Task - Genuine *vs.* Sham	0,02	-0,003	0,042	0.05	21.719
SMR	Cz	Trial:Task - Sham *vs.* Passive	-0,025	-0,047	-0,002	0.124	0.015
Beta	Cz	Trial	0,005	-0,007	0,017	0.009	4.155
Beta	Cz	Task - Genuine *vs.* Sham	-0,038	-0,486	0,411	0.226	0.762
Beta	Cz	Task - Sham *vs.* Passive	0,065	-0,379	0,508	0.234	1.591
Beta	Cz	Trial:Task - Genuine *vs.* Sham	0,032	0,003	0,061	0.16	63.544
Beta	Cz	Trial:Task - Sham *vs.* Passive	-0,031	-0,059	-0,002	0.133	0.019
**Theta**	**Pz**	**Trial**	**0,029**	**0,016**	**0,041**	**6484.6**	***Inf.***
Theta	Pz	Task - Genuine *vs.* Sham	0,256	-0,263	0,77	0.436	5.148
Theta	Pz	Task - Sham *vs.* Passive	-0,22	-0,726	0,289	0.373	0.242
Theta	Pz	Trial:Task - Genuine *vs.* Sham	0,012	-0,019	0,044	0.022	3.581
Theta	Pz	Trial:Task - Sham *vs.* Passive	-0,016	-0,048	0,015	0.027	0.173
**SMR**	**Pz**	**Trial**	**0,018**	**0,009**	**0,027**	**9.546**	**12635.36**
SMR	Pz	Task - Genuine *vs.* Sham	0,168	-0,319	0,65	0.311	3.1
SMR	Pz	Task - Sham *vs.* Passive	-0,056	-0,53	0,424	0.244	0.688
SMR	Pz	Trial:Task - Genuine *vs.* Sham	0,017	-0,006	0,039	0.032	12.377
SMR	Pz	Trial:Task - Sham *vs.* Passive	-0,021	-0,043	0,001	0.065	0.032
Beta	Pz	Trial	0	-0,011	0,011	0.006	1.085
Beta	Pz	Task - Genuine *vs.* Sham	-0,058	-0,507	0,391	0.233	0.66
Beta	Pz	Task - Sham *vs.* Passive	0,057	-0,386	0,498	0.229	1.517
Beta	Pz	Trial:Task - Genuine *vs.* Sham	0,014	-0,014	0,041	0.023	5.315
Beta	Pz	Trial:Task - Sham *vs.* Passive	-0,025	-0,052	0,002	0.075	0.033

Each model reported has been computed twice in order to ensure the stability of the BFs. If not specified, each numerical value corresponds to the average of the values obtained across these two model computations. The 'Estimate' column stands for the averaged posterior group-level effects (slopes) of each model 'Parameter' (in z-score standardised units). For the 'Trial' predictor, the estimate corresponds to the group-level effect of one trial within the Genuine group (defined as reference for subsequent comparisons for the Task predictor). For the 'Task' predictor, the estimate of both comparisons ('Genuine *vs.* Sham' and 'Sham *vs.* Passive') refers to the between-group effect within the first trial of the transfer block (defined as reference for subsequent comparisons for the Trial predictor). The 'Lower' and 'Upper' columns correspond to the minimal lower and maximal upper bounds of the two 95% CrI computed. The 'BF_{10}' and 'BF_{10+}' columns correspond to the BF in favour of the alternative hypothesis (relative to the null) and the positive directional (i.e., one-sided) BF, respectively.
Lines in gold highlight the EEG features for which BFs quantify sufficient evidence in favour of the alternative hypothesis over the null (i.e., presence of an effect).

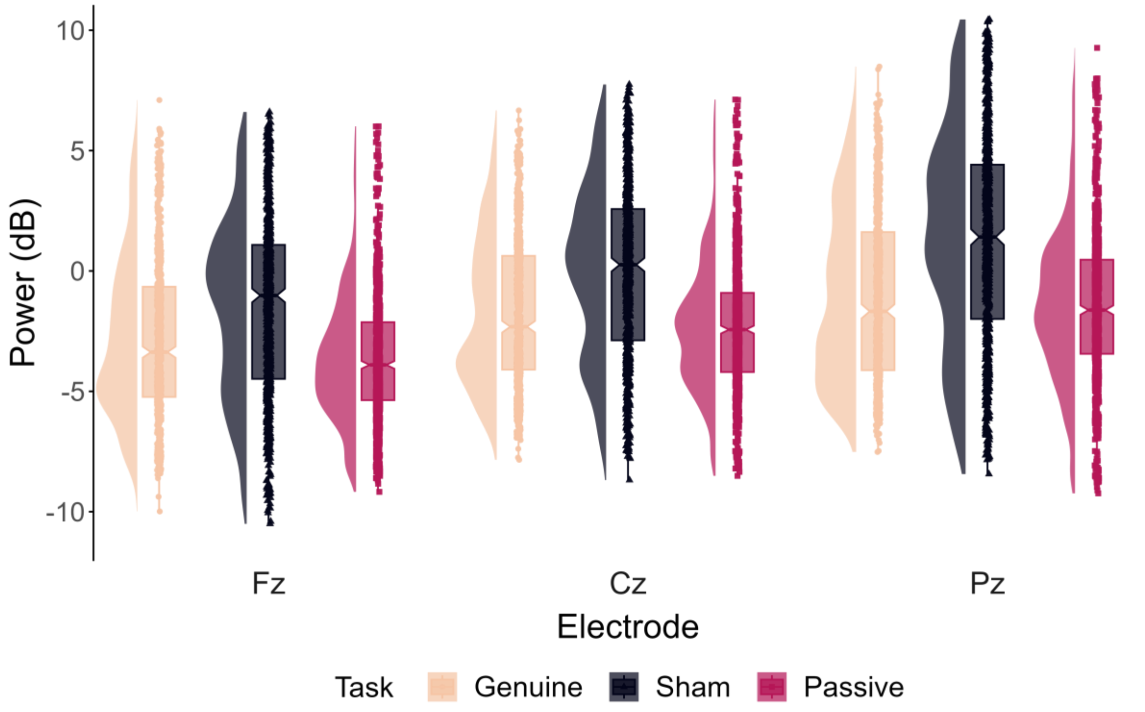

Supplementary Figure 1 Alpha power levels throughout the session in function of group allocation. Distributions of individual alpha (8-12 Hz) spectral power during the experimental session. Each point represents the alpha power computed on one trial (all blocks confounded) and for one participant, depending on the Task participants were submitted to (cream: Genuine EEG-NF; dark blue: Sham EEG-NF; pink red: Passive visualisation task) and electrode position (left: Fz; middle: Cz; right: Pz). Each boxplot represents the median, the first and the third quartiles, along with the 95% confidence interval of the resulting distribution (for the Genuine and Sham groups, n = 960, i.e., 30 participants * 32 trials; for the Passive group: n = 1024, i.e., 32 participants * 32 trials). Visually, we can acknowledge an overall higher alpha power in the Sham group (*vs*. Genuine and Passive groups) at each electrode position, even though Bayesian evidence was insensitive for between-group differences (see Supplementary Tables 7-8).
